# Supplementary material for: The causal role of circulating inflammatory markers in osteoporosis: a bidirectional Mendelian randomized study
Source: Front Immunol. 2024 Jul 18;15:1412298. doi: 10.3389/fimmu.2024.1412298 (PMC11291241; doi:10.3389/fimmu.2024.1412298)
Supplement: Supplementary file 1 [file DataSheet_1.zip › supplementary material/OSTEOPOROSIS-CIM/GCST90274847.tsv.gz_forest.pdf]

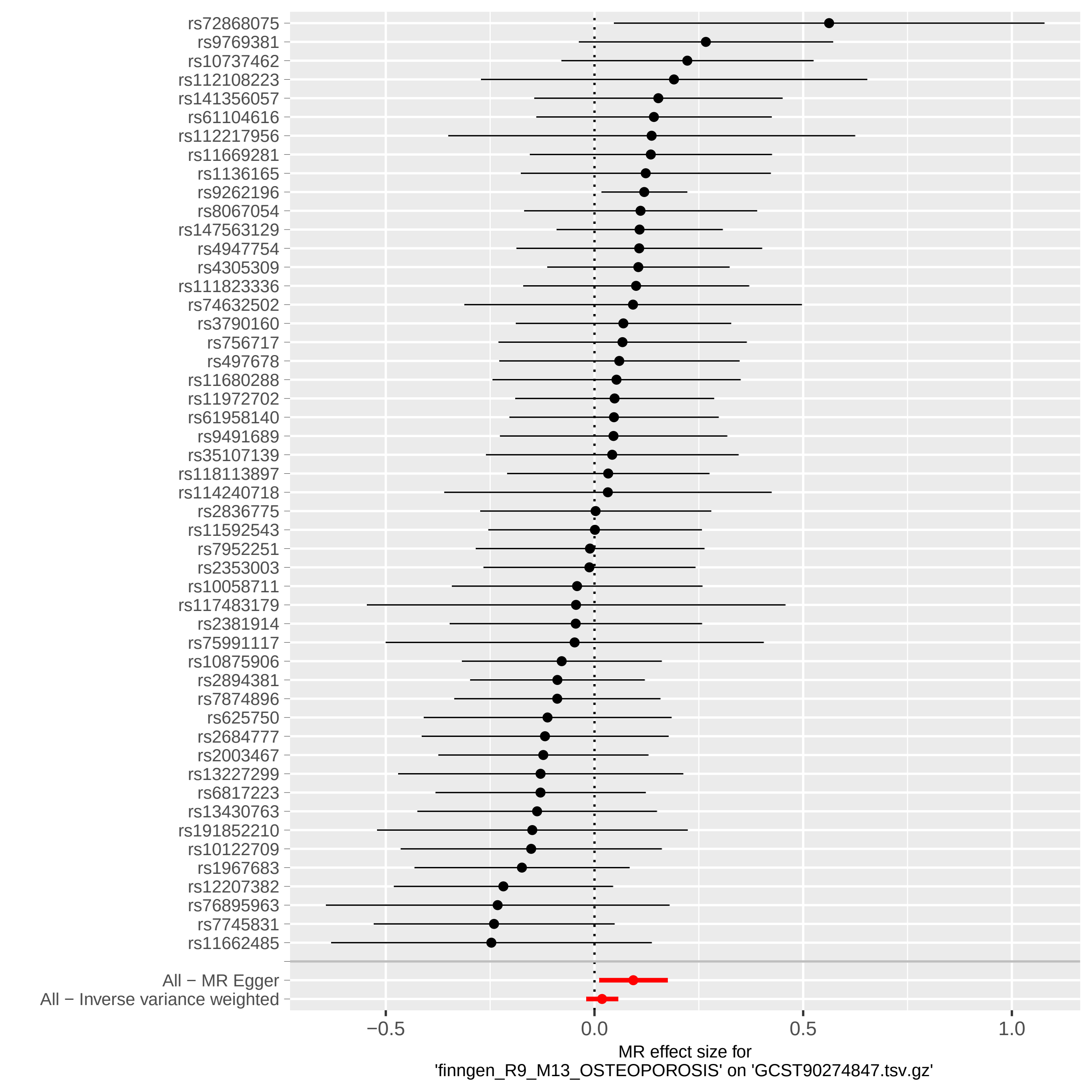
rs72868075
rs9769381
rs10737462
rs112108223
rs141356057
rs61104616
rs112217956
rs11669281
rs1136165
rs9262196
rs8067054
rs147563129
rs4947754
rs4305309
rs111823336
rs74632502
rs3790160
rs756717
rs497678
rs11680288
rs11972702
rs61958140
rs9491689
rs35107139
rs118113897
rs114240718
rs2836775
rs11592543
rs7952251
rs2353003
rs10058711
rs117483179
rs2381914
rs75991117
rs10875906
rs2894381
rs7874896
rs625750
rs2684777
rs2003467
rs13227299
rs6817223
rs13430763
rs191852210
rs10122709
rs1967683
rs12207382
rs76895963
rs7745831
rs11662485
All − MR Egger
All − Inverse variance weighted
−0.5
0.0
0.5
1.0
MR effect size for
'finngen_R9_M13_OSTEOPOROSIS' on 'GCST90274847.tsv.gz'
